# Certain Tomato Root Exudates Induced by *Pseudomonas stutzeri* NRCB010 Enhance Its Rhizosphere Colonization Capability

**DOI:** 10.3390/metabo13050664

**Published:** 2023-05-16

**Authors:** Huanhuan Zhang, Donghui Zheng, Chun Hu, Wenwen Cheng, Peng Lei, Hong Xu, Nan Gao

**Affiliations:** 1 School of Biotechnology and Pharmaceutical Engineering, Nanjing Tech University, Nanjing 211816, China; 2 College of 2011, Nanjing Tech University, Nanjing 211816, China; 3 School of Food Science and Light Industry, Nanjing Tech University, Nanjing 211816, China

**Keywords:** PGPR, *Pseudomonas* sp., chemotaxis, biofilm, colonization, n-hexadecanoic acid

## Abstract

Plant growth-promoting rhizobacteria (PGPR) can colonize plant root surfaces or form biofilms to promote plant growth and enhance plant resistance to harsh external environments. However, plant–PGPR interactions, especially chemical signaling molecules, are poorly understood. This study aimed to gain an in-depth understanding of the rhizosphere interaction mechanisms between PGPR and tomato plants. This study found that inoculation with a certain concentration of *Pseudomonas stutzeri* significantly promoted tomato growth and induced significant changes in tomato root exudates. Furthermore, the root exudates significantly induced NRCB010 growth, swarming motility, and biofilm formation. In addition, the composition of the root exudates was analyzed, and four metabolites (methyl hexadecanoate, methyl stearate, 2,4-di-tert-butylphenol, and n-hexadecanoic acid) significantly related to the chemotaxis and biofilm formation of NRCB010 were screened. Further assessment showed that these metabolites positively affected the growth, swarming motility, chemotaxis, or biofilm formation of strain NRCB010. Among these, n-hexadecanoic acid induced the most remarkable growth, chemotactic response, biofilm formation, and rhizosphere colonization. This study will help develop effective PGPR-based bioformulations to improve PGPR colonization and crop yields.

## 1. Introduction

Plant growth-promoting rhizobacteria (PGPR) have been widely used in agricultural applications because of their safety, biological control of diseases and insect pests, and ability to induce systemic tolerance [1]. The ability of PGPR to colonize the surface or inside plant roots is a prerequisite for the interaction between microorganisms and plants. It is also an essential condition for probiotics [1,2]. Chemotaxis and biofilm formation are part of the colonization mechanism of the bacterial root surface and are two major steps for establishing microorganisms in the rhizosphere [2,3]. Bacterial chemotaxis involves bacteria being induced and recruited by specific substances in root exudates to approach and gather around plant roots, an essential premise for microorganisms to colonize the root surface [4] and enhance the ability of bacteria to colonize plant host roots [2,5]. Biofilm formation is also a key factor affecting the colonization ability of PGPR [6,7]. Stable biofilm formation on the surface of plant roots is a visual indication of successful PGPR colonization. Biofilms enhance the resistance of microorganisms to adverse environments such as drought, extreme pH, and toxic substances in the soil, thereby maintaining high populations [6,8]. Chemotaxis, swarming motility, and biofilm formation are three crucial activities for PGPR to successfully colonize plant roots [3,9].

Colonization, chemotaxis, and biofilm formation by PGPR are induced by the root exudates and certain metabolites [3,5,10]. Root exudates are composed of low-molecular-weight carbon compounds that are passively or actively exuded from plant roots. Malic and citric acids play crucial roles in attracting and initiating the PGPR colonization of host roots [7], and tryptophan improves the colonization ability of *Bacillus amyloliquefaciens* SQR9 in cucumber roots [11]. Organic acids (such as malic acid, citric acid, and succinic acid), amino acids (such as methionine, glutamic acid, and alanine), and phenolic acids (such as benzoic acid) induce chemotaxis and swarming motility [12,13,14]. In addition, palmitic acid, 2-methylbutyric acid, stearic acid, and oleic acid favor PGPR mobility, chemotaxis, and attachment to the roots [15]. Furthermore, 2,4-di-tert-butylphenol and methyl stearate affect the growth and chemotaxis of microorganisms [10,16].

Environmental factors change root exudate metabolites. Temperature, light, soil nutrients, and microorganisms affect the composition and quantity of root exudates [12,17,18]. Among them, rhizobacteria are important in modifying and limiting root exudates through their metabolic activities by decomposing and transforming them [12,19]. The root exudate metabolite profile changes owing to the presence of microorganisms. For example, when PGPR colonize the plant rhizosphere, root exudate metabolites such as organic acid (benzoic acid and salicylic acid), flavonoids (quercetin and biochanin A), and amino acids (glycine, tryptophan, and glutamine) increase [11,12,13,14,20].

In summary, PGPR change the root exudate metabolite profile; conversely, root exudate metabolites regulate the colonization, chemotaxis, and biofilm formation of PGPR, which optimizes PGPR survival and function. *P. stutzeri* NRCB010 (NRCB010) was previously isolated from a rice rhizosphere soil [21]. For further development and utilization of NRCB010, the interactions between NRCB010 and host plants, that is, the effects of NRCB010 on tomato root exudate metabolite profiles and the subsequent effects of root exudate metabolites on NRCB010 biomass, colonization, chemotaxis, and biofilm formation, were investigated.

## 2. Materials and Methods

### 2.1. PGPR Strain and Plant Materials

*P. stutzeri* NRCB010 (NRCB010; GenBank accession number MZ165017) was isolated from rice rhizosphere soil, Yixing (31°12′ N; 119°52′ E), Jiangsu Province, China [22], and stored in a Luria–Bertani (LB) liquid medium with 30% glycerol at −80 °C. To induce NRCB010 fermentation, a single colony was cultured in the LB liquid medium overnight at 30 °C and 200 rpm unless specified otherwise. The resulting fermentation product was diluted to approximately OD600 = 1.0 (about 10^8^ CFU ml^−1^) for further use.

Tomato (*Solanum lycopersicum* L. cv. Xinzhongshusihao) was purchased from Xingke Seed Co., Ltd., (Tianjin, China). The seeds were surface-sterilized in 2.5% (*w*/*v*) sodium hypochlorite for 10 min and washed ten times with sterilized distilled water. The seedlings were cultured in a greenhouse controlled at 25 ± 2 °C, under a 14 h/10 h (light/dark) photoperiod.

### 2.2. Effects of NRCB010 on Tomato Growth in Hydroponic Conditions

Single NRCB010 colonies were cultured in NBNS (highly peptone 5 g L^−1^, LAB-Lemco powder 3 g L^−1^, sodium nitrate 25 mg L^−1^, and sodium succinate 710 mg L^−1^, pH 7.0) overnight. The overnight NRCB010 fermentation culture was centrifuged at 6000× *g* for 5 min and resuspended in one-tenth of the Hoagland solution.

Surface-sterilized seeds were germinated in a hydroponic system with one-tenth Hoagland solution. After seven days of growth, uniform seedlings were cultured with one-tenth Hoagland solution containing different concentrations of NRCB010 (0 (control), 10^3^, 10^5^, and 10^7^) for four days. Thereafter, the seedlings were transferred to one-tenth Hoagland solution without NRCB010, and the solution was renewed every four days. Each treatment was performed in quadruplicates.

Seedlings were harvested 14, 18, and 22 days after inoculation (DAI). The plant height was measured using a ruler, and the stem diameter was measured using a Vernier caliper. Shoot and root fresh weights were measured immediately after collection, and dry weights were measured after oven-drying at 65 °C to a constant weight.

### 2.3. Tomato Root Exudates Collection and Component Analysis

#### 2.3.1. Root Exudates Collection and Component Measurements

Root exudates were collected as previously described [15,23] with minor modifications. Four uniform 7-day seedlings were inoculated with 0 (NRCB010−) and 10^5^ CFU mL^−1^ (NRCB010+) NRCB010. Three DAI, the roots were washed five times with sterile water, followed by ultrasound treatment at 100 Hz for 5 min, and then washed once with sterile water. Four treated seedlings were transplanted into 60 mL of ultrapure water to immerse the roots, grown for 24 h, and then removed. The crude root exudates were collected, centrifuged at 6500× *g* for 5 min to remove cell debris, and sterilized using a 0.22 μm syringe filter (Millipore Corp, Billerica, MA, USA). Collected exudates were normalized to 300 mL g^−1^ root fresh weight. Then soluble sugar, protein, and free amino acid concentration in the normalized root exudates were measured. The root exudate soluble sugar concentration was determined by Coomassie brilliant blue G-250 staining. The soluble protein concentration was determined using the ninhydrin color development method. The free amino acid concentration was determined using the anthrone method.

#### 2.3.2. Root Exudates Component Identification

For root exudate component identification, the normalized root exudates were further lyophilized, dissolved in methanol to concentrate 10 times, and stored at −80 °C until use. Two milliliter root exudates were derived with 10 μL N, O-bis (trimethylsilyl) trifluoroacetamide (BSTFA: TMC = 5:1) at 60 °C for 1 h [23]. The residual solution was sterilized using a 0.22 μm syringe filter (Millipore, USA) and stored at −80 °C for component analysis. Detection instrument: gas chromatography/mass spectrometry (GC-MS) AgilENT-6890N/59731 (Thermo, Riva del Garda, Italy); chromatographic conditions: HP-5MS (30 m × 0.25 mm × 0.25 μm); carrier gas: high-purity helium; column flow rate: 1.0 mL min^−1^; injection volume: 1 μL; solvent delay: 3 min; injection mode: no shunt; injector temperature: 280 °C; tank temperature: kept at 70 °C for 2 min, then increased from 70 °C to 300 °C for 10 min at a speed of 10 min^−1^; ion source energy of MS detector: 70 eV; scanning range: 35–650 amu; scanning period: 0.32. Compound hits were identified by searching the extracted mass spectra from the above steps in the National Institute of Standards and Technology (NIST) library using NIST MS search v. 2.0 (Agilent, Santa Clara, CA, USA).

### 2.4. Effects of Root Exudates and Certain Metabolites on NRCB010 Growth

#### 2.4.1. Root Exudates and Certain Metabolites Preparation

Root exudates were diluted five times with methanol. Next, certain metabolites were dissolved with 100% ethanol to, 1, 5, and 10 g L^−1^.

##### NRCB010 Biomass

NRCB010 was cultured in a one-fifth NBNS liquid medium. Two microliter NRCB010 diluted solution, and 10 μL diluted root exudates or 10 μL NRCB010 diluted solution, and 2 μL certain metabolite solutions were thoroughly mixed with 188 μL one-fifth NBNS liquid medium, and added into a well of a 96-well microtiter microplate. Equal volumes of methanol and ethanol were used as controls to substitute for the root exudates and metabolite solutions, respectively. The mixture was cultured at 100 rpm for 48 h. The OD600 value, indicating NRCB010 biomass, was measured using a microplate analyzer (INFINITE 200 PRO, Grödig, Austria).

##### Diameter of NRCB010 Swarming Area

Semi-solid LB plates (0.5% agar) containing 5% (*v*/*v*) root exudates or 1% (*v*/*v*) metabolite solution were used for the swarming assay [9]. Equal volumes of methanol and ethanol were used as controls to substitute for the root exudates and metabolite solutions, respectively. A 4 mm diameter sterile filter paper disk was placed at the center of the plate. Two microliter of the NRCB010 diluted solutions were pipetted onto the paper disk. The diameter of the swarming area was measured after static incubation for 24 h for the root exudate treatment and 72 h for the metabolite treatment.

##### Biofilm Formation

The diluted NRCB010 fermentation was centrifuged at 6500× *g* for 5 min, washed with 0.86% (*w*/*v*) NaCl, and resuspended in the same-volume minimal lactate-containing medium (medium K) [8,9]. About 37.5 μL of the bacterial solution and 112.5 μL of medium K containing root exudates or metabolite solutions were mixed and added into a well of a 96-well microtiter microplate. The final concentrations of root exudates and metabolite solutions were 5% (*v*/*v*) and 1% (*v*/*v*), respectively. Equal volumes of methanol and ethanol were used as controls to substitute for the root exudates and metabolite solutions, respectively. Each treatment was performed in triplicates, and the biological replicates contained seven technical replicates. After incubation for 48 h, NRCB010 biofilm formation was determined using crystal violet staining.

##### Chemotaxis

Quantitative chemotaxis was measured using a capillary assay as previously described [9]. Briefly, the diluted NRCB010 fermentation was centrifuged at 6500× *g* for 5 min and resuspended with the same volume of 0.86% (*w*/*v*) NaCl. A 200 μL pipette tip was used as a chamber for holding 100 μL of NRCB010 resuspension. A 4-cm 25-gauge needle, used as the chemotaxis capillary, was attached to a 1 mL disposable sterile syringe. A 100 μL portion of 0.86% of NaCl containing 1% (*v*/*v*) metabolite solutions to be tested was drawn up through the needle into the syringe (needle-syringe capillary). Equal volumes of ethanol were used as a substitute for the metabolite solutions as controls. After static culture 2 h at 30 °C in the dark, the needle syringe was removed from the NRCB010 resuspension contained in the pipette tip, and the contents were diluted in 0.86% (*w*/*v*) NaCl. The dilutions were then plated onto an LB medium. Chemotaxis was calculated from the colony forming units (CFUs) on the plates after incubation at 36 h at 30 °C.

##### Colonization

Colonization ability was measured as previously described [21] with minor modifications. Briefly, the diluted NRCB010 fermentation was centrifuged at 6500× *g* for 5 min and resuspended in a 10-time volume of 0.86% (*w*/*v*) NaCl. The surface-sterilized tomato seeds were germinated on a water agar plate for seven days. Then uniform seedlings were immersed in a bacterial suspension with 0 or 50 mg L^−1^ metabolite for 2 h. After that, the seedlings were removed from the solution and drained of excess water on the surface, followed by being cultured on a one-fifth MS medium containing 0.8% agar and 0.6% sucrose for two days. The tomato roots were cut off; lightly washed with sterile water to remove uncolonized bacteria; weighed; ground; diluted with 0.86% (*w*/*v*) NaCl to 10^−2^, 10^−3^, and 10^−4^; and finally coated onto the LB medium. Colonization ability was calculated from the CFUs on the plates after incubation at 48 h at 30 °C.

### 2.5. Statistical Analyses

Hierarchical cluster analysis was performed using the “vegan and pheatmap” package in R (version 3. 5.0, https://www.r-project.org/, access on 1 April 2018) by setting the Euclidean distance as the similarity measure and Ward’s linkage as the clustering algorithm. Pearson’s correlation analysis, one-way analysis of variance (ANOVA), independent samples *t*-test (*p* < 0.05), and Duncan’s multiple range test (*p* < 0.05) were performed using SPSS Statistics (version 26.0, 1 May 2019, IBM., New York, NY, USA).

## 3. Results

### 3.1. Effects of NRCB010 on Tomato Growth in Hydroponic Conditions

**Figure 1:**
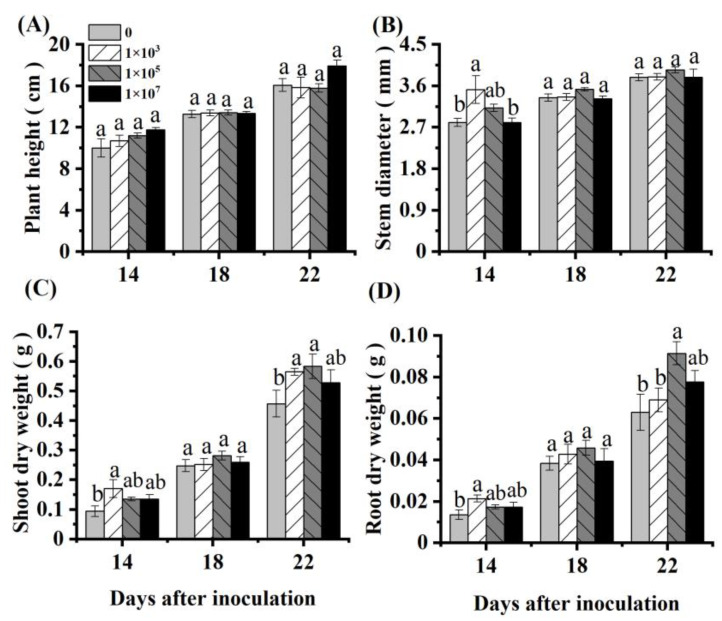
Effects of *Pseudomonas stutzeri* NRCB010 on tomato growth in hydroponic conditions. (**A**) Plant height; (**B**) stem diameter; (**C**) shoot dry weight; (**D**) root dry weight. Values are means ± standard error (n = 4). Different letters above the bars at the same days after inoculation denote significant differences between inoculation concentrations (CFU) by Duncan’s post hoc test (*p* < 0.05).

Inoculation with an appropriate concentration of NRCB010 increased the tomato biomass (Figure 1). Compared with control, the shoot and root dry weight of the tomato significantly increased by 27.7% and 45.2%, respectively, at 22 DAI with 10^5^ CFU mL^−1^ NRCB010 (Figure 1C,D). Within 22 DAI, the plant height and stem diameter did not significantly change (Figure 1A,B).

#### Effects of NRCB010 on Tomato Root Exudate (RE) Component

Compared with the soluble sugar concentration of root exudates without NRCB010 treatment, the soluble sugar concentration of those with NRCB010+ treatment was significantly decreased; on the contrary, the soluble protein concentration was significantly increased (Table 1).

### 3.2. Effects of Root Exudates on NRCB010 Growth

The NRCB010 biomass, swarming area diameter, and biofilm formation were significantly increased by the tomato root exudates (Table 2). Compared with those of NRCB010−-treated root exudates, the NRCB010 biomass, diameter of the swarming area, and biofilm formation of NRCB010+-treated root exudates were significantly increased by 15.6%, 8.0%, and 16.9%, respectively (Table 2).

### 3.3. Changes in Tomato Root Exudate Profile in Response to NRCB010

**Figure 2:**
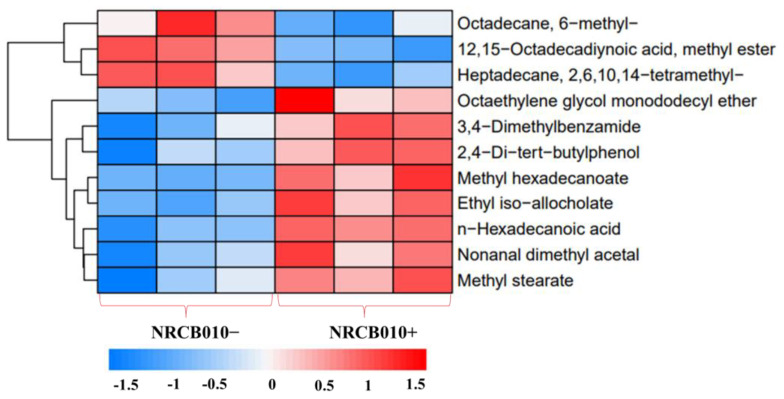
Global different metabolite profiles of tomato root exudate (NRCB010−) and induced by *Pseudomonas stutzeri* NRCB010 (NRCB010+). Euclidean distance similarity metric of NRCB010− and NRCB010+ metabolites, and complete linkage analysis. Each row represents the relative concentration levels of the labeled metabolite in color, according to the scale at the bottom of the figure.

Forty-one root exudate components were detected in both the NRCB010− and NRCB010+ treatments (Table S1) using GC-MS. The relative contents of 11 components were significantly changed between NRCB010− and NRCB010+ treatments (Figure 2 and Table 3). Compared with those of NRCB010−-treated root exudates, the relative contents of eight components of NRCB010+-treated root exudates were significantly increased (*p* < 0.05), and the relative contents of the three components of NRCB010+-treated root exudates were significantly decreased (*p* < 0.05) (Table 3).

To focus on specific root exudate metabolites, pearson correlation analysis was conducted between the relative content of the tomato root exudate components (original data in Table 3) and the biomass, diameter of the swarming area, and biofilm formation of NRCB010 (original data in Table 2). Among the 11 root exudate components, the relative contents of 2, 6, and 5 components were significantly positively correlated with the biomass, diameter of the swarming area, and biofilm formation, respectively (Table 4).

Based on chemical safety information and related references, four metabolites, 2,4-di-tert-butylphenol, methyl hexadecanoate, n-hexadecanoic acid, and methyl stearate, were selected for further analysis, and the solute’s peak positions on ion chromatogram are shown in Figure S2.

### 3.4. Effects of Four Root Exudate Metabolites on NRCB010 Growth

**Figure 3:**
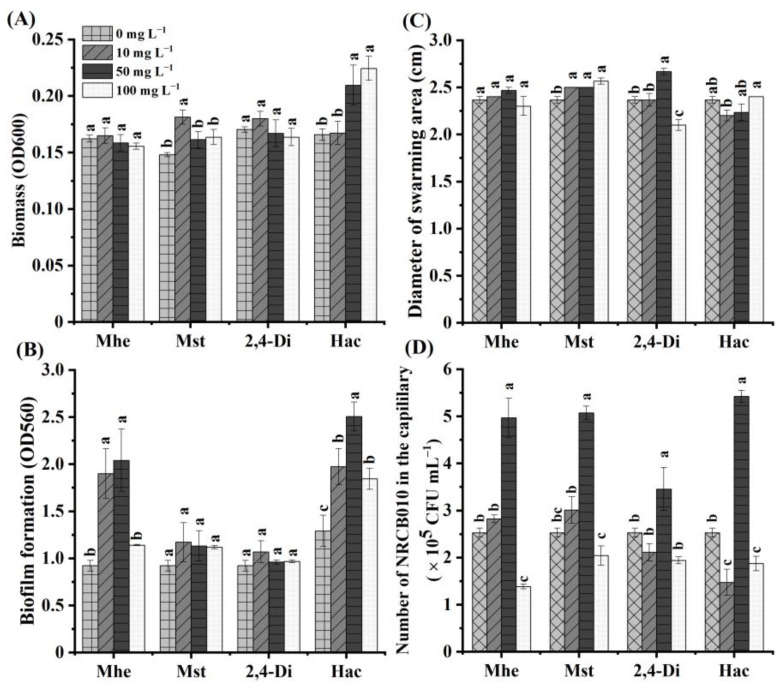
Effects of four tomato root exudate metabolites on *Pseudomonas stutzeri* NRCB010. (**A**) Biomass (n = 8), (**B**) biofilm formation (n = 8), (**C**) diameter of swarming area (n = 3), and (**D**) chemotaxis (n = 3). Bars are means ± standard error. Different letters above the bars at the same metabolite denote significant differences between concentrations by Duncan’s post hoc test (*p* < 0.05). Mhe presents methyl hexadecanoate; Mst presents methyl stearate; 2,4-Di presents 2,4-di-tert-butylphenol; and Hac presents n-hexadecanoic acid.

**Figure 4:**
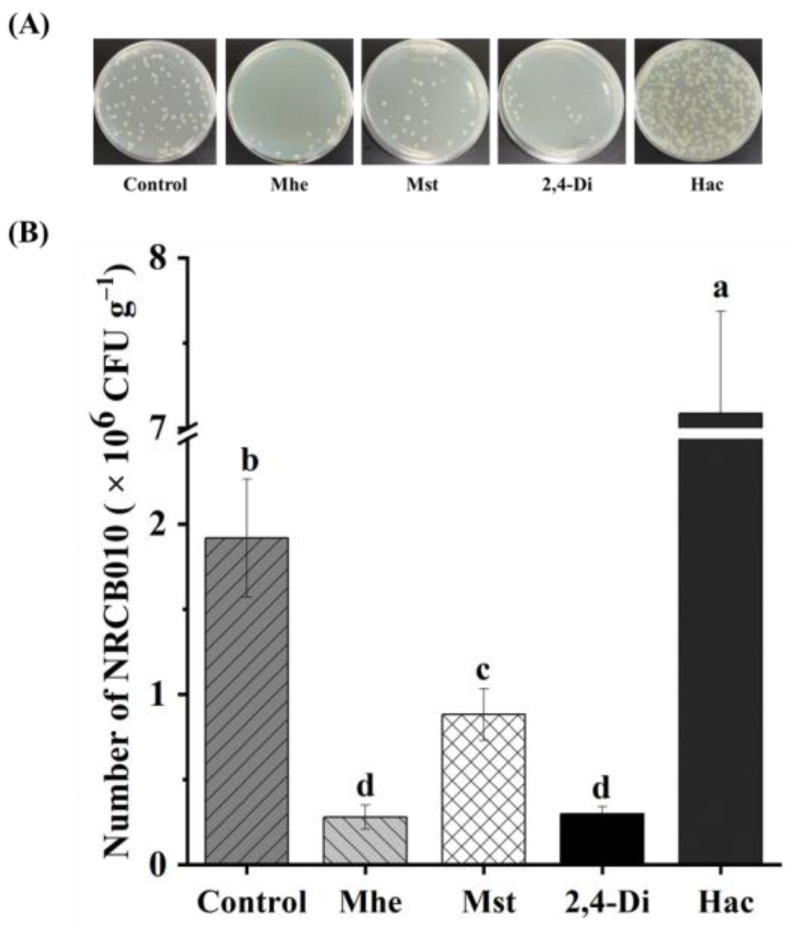
Effects of four tomato root exudate metabolites on *Pseudomonas stutzeri* NRCB010 colonization on tomato root surface. (**A**) Representative image of CFUs on spread plate. (**B**) Number of NRCB010 colonized on root surface. Bars are means ± standard error (n = 3). Different letter above the bars denotes significant differences between metabolites by Duncan’s post hoc test (*p* < 0.05). Mhe presents methyl hexadecanoate; Mst presents methyl stearate; 2,4-Di presents 2,4-di-tert-butylphenol; and Hac presents n-hexadecanoic acid.

Four metabolites, namely methyl hexadecanoate, methyl stearate, 2,4-di-tert-butylphenol, and n-hexadecanoic acid, were selected to test their effects on NRCB010 growth (Figure 3). Compared with no metabolite control, the NRCB010 biomass was significantly increased by 10 mg L^−1^ methyl stearate and 50 and 100 mg L^−1^ n-hexadecanoic acid (Figure 3A); biofilm formation was significantly increased by 10 and 50 mg L^−1^ methyl hexadecanoate and n-hexadecanoic acid (Figure 3B); the diameter of the swarming area was significantly increased by methyl stearate and 50 mg L^−1^ 2,4-di-tert-butylphenol, and significantly decreased by 100 mg L^−1^ 2,4-di-tert-butylphenol (Figure 3C); chemotaxis was significantly increased by 50 mg L^−1^ methyl hexadecanoate, 50 mg L^−1^ methyl stearate, 50 mg L^−1^ 2,4-di-tert-butylphenol, and 50 mg L^−1^ n-hexadecanoic acid, and significantly decreased by 100 mg L^−1^ methyl hexadecanoate, 100 mg L^−1^ methyl stearate, and 100 mg L^−1^ n-hexadecanoic acid (Figure 3D); the number of NRCB010 colonized on root surface was significantly increased by 50 mg L^−1^ n-hexadecanoic acid and significantly decreased by 50 mg L^−1^ methyl hexadecanoate, 50 mg L^−1^ methyl stearate, and 50 mg L^−1^ 2,4-di-tert-butylphenol (Figure 4A,B).

## 4. Discussion

Root exudates contain various metabolites, including organic acids, amino acids, sugars, and phenolic compounds [12,13]. There are different methods to collect root exudates such as hydroponic growth and sampling, soil growth–hydroponics sampling, and soil growth–rhizobox sampling [13,14,24]. To avoid soil components affecting the root metabolites [25], the hydroponic growth and sampling approach was used to collect root exudates [24]. Rhizobacteria change their root exudate profile, utilize root exudates as a source of carbon and energy, promote and protect growth, and increase the subsequent yield of the host plant [26]. *Ph. liquidambaris* B3 inoculation altered the concentrations of phenolic acids, flavonoids, organic acids, and amino acids in peanut root exudates [13]. *Pseudomonas* sp. RP2 in groundnut roots changed the contents of myristic acid, stearic acid, and palmitic acid in root exudates [12]. In this study, NRCB010 altered the relative contents of tomato metabolites (Table S1), which are similar to the previous results. Compared with uninoculated plants, *Giomus fasciculatum* increased protein and the phenolic substance contents of *Leucaena leucmepluzla* root exudates while decreasing sugar (mainly glucose) [27]. In this study, NRCB010 increased the soluble protein concentration and reduced the soluble sugar concentration in tomato root exudates (Table 1). Soluble sugars in root exudates can be used as carbon sources to provide nutrients for the growth of rhizosphere soil microorganisms [2,7]; thus, the concentration of soluble sugars decreases. When tomato root exudate was added, *B. amyloliquefaciens* T-5 and *Ph. liquidambaris* B3 rapidly grew [7,13]. In this study, although the soluble sugar concentration decreased, the NRCB010 biomass formation was not significantly changed by root exudates from the NRCB010− and NRCB010+ treatments (Table 2). These results indicated that NRCB010 might induce the “favorable” carbon source and restrain the “unfavorable” carbon source as the “carbon catabolite repression effect” on the carbon utilization [28].

PGPR change the root exudate metabolites. *Ph. liquidambaris* B3 changed the secretion pattern of peanut root exudates by increasing the concentrations of specific components, including organic acid, amino acid, phenolic acid, and flavonoid, rather than by inducing the production of different components [13]. The metabolite profile of groundnut root exudates significantly changed after inoculation with *B. sonerensis* RS4 or *P. aeruginosa* RP2 [14]. NRCB010 altered the secretion pattern of root exudates by increasing the concentration of specific components rather than inducing different components’ production (Table 3 and Table S1). That is to say, the metabolites were secreted by a tomato, and NRCB010 induced the changes of relative contents. In this study, the relative contents of 11 components were significantly changed between the NRCB010− and NRCB010+ treatments (Table 3), including 2,4-di-tert-butylphenol, methyl hexadecanoate, methyl stearate, and n-hexadecanoic acid. *P. aeruginosa* RP2 also increases the N-hexadecanoic acid content in the groundnut root exudates [12].

Root exudates change the PGPR growth, swarming, and biofilm formation. Compared with the root exudates without inoculation, the root exudates of peanut treated with *P. aeruginosa* P4 had stronger chemotaxis and colonization ability to P4 and *Bradyrhizobium* [20]. Watermelon root exudates significantly induced the chemotaxis, swarming motility, and biofilm formation of *Bacillus amyloliquefaciens* TR2 [9]. Root exudates strongly induced the swarming and biofilm formation of NRCB010 compared with the control. Those of the NRCB010+ treatment were significantly higher than those of the NRCB010− treatment (Table 2), suggesting that these root exudates may further improve the colonization efficiency of NRCB010.

Certain root exudate metabolites change the PGPR growth, swarming, and biofilm formation. 2,4-Di-tert-butylphenol and methyl stearate showed strong chemotaxis in the rhizosphere bacteria of *Casuarina equisetifolia* L. [10]. In this study, methyl stearate and 2,4-di-tert-butylphenol promoted NRCB010 swarming (Figure 3C), which confirms previous results. In the presence of organic acids from banana root exudates, *B. amyloliquefaciens* NJN-6 increases chemotaxis and biofilm formation [29]. Methyl hexadecanoate also promoted the chemotaxis and biofilm formation of NRCB010 (Figure 3B,D). However, 50 mg L^−1^ 2,4-di-tert-butylphenol, methyl hexadecanoate, and methyl stearate inhibited NRCB010 colonization (Figure 4), indicating that the appropriate concentration of these metabolites to promote colonization should be further investigated. N-hexadecanoic acid from *Limonium sinense* root exudates promotes chemotaxis and growth of *B. flexus* KLBMP 4941 [15]. In this study, n-hexadecanoic acid promoted the NRCB010 growth, motility, biofilm formation, and colonization of tomato root surfaces (Figure 3A,B,D and Figure 4) and alleviated the NRCB010 growth under environmental stress (Figure S1). These results showed that NRCB010 increased tomato root exudate metabolites to provide sufficient nutrients for bacterial growth, motility, and biofilm formation. n-Hexadecanoic acid is one of the main chemoattractants that induces the recruitment of NRCB010 to the tomato rhizosphere, thereby effectively promoting the colonization of the tomato rhizosphere strain. The effects of n-hexadecanoic acid on bacterial motility mechanisms, such as swimming, twitching, gliding, and sliding, and how it can affect the modulation of colonization should be further studied.

## 5. Conclusions

Colonization of the plant rhizosphere is a prerequisite for plant growth-promoting rhizobacteria (PGPR) function, and plant root exudates play an essential role in regulating plant–microbe interactions. NRCB010 promoted tomato growth under hydroponic conditions, and the tomato root exudates significantly increased the NRCB010 biomass, swarming area diameter, and biofilm formation. In addition, NRCB010 changed tomato root exudate components, and the relative content of 11 components significantly changed. Among these, methyl hexadecanoate, methyl stearate, and 2,4-di-tert-butylphenol increased the NRCB010 swarming, chemotaxis, and biofilm formation at proper concentrations but inhibited NRCB010 colonization on the tomato root surface at the tested concentrations; n-hexadecanoic acid increased NRCB010 chemotaxis, biofilm formation, and colonization of the tomato root surface; thus, these metabolites may play a key role in facilitating root colonization.

## 6. Patents

Part work reported in this manuscript was used for China patent (application No. 202310527642.6).

## Figures and Tables

**Table 1:** Effect of *Pseudomonas stutzeri* NRCB010 on tomato root exudate (RE) component.

Treatment	Soluble Sugar (mg g^−1^)	Soluble Protein (mg g^−1^)	Free Amino Acids (mg g^−1^)
RE/NRCB010−	8.1 ± 1.5 **	66.5 ± 4.0	0.93 ± 0.06
RE/NRCB010+	4.7 ± 0.4	126.4 ± 29.0 **	0.97 ± 0.07

Note: The asterisks after the data of the same column mean significant differences between treatments by *t* test. ** *p* < 0.01.

**Table 2:** Effects of tomato root exudates (RE) on *Pseudomonas stutzeri* NRCB010 growth.

Treatment	Biomass (OD600)	Diameter of Swarming Area (cm)	Biofilm Formation (OD560)
Methanol control	0.10 ± 0.01 ^b^	1.57 ± 0.03 ^c^	1.14 ± 0.08 ^c^
RE/NRCB010−	0.13 ± 0.00 ^ab^	1.67 ± 0.03 ^b^	1.92 ± 0.12 ^b^
RE/NRCB010+	0.15 ± 0.01 ^a^	1.80 ± 0.00 ^a^	2.24 ± 0.03 ^a^

Note: Different letters after the data of the same column mean significant differences between treatments by Duncan’s post hoc tests (*p* < 0.05).

**Table 3:** Significant difference compositions between tomato root exudates (NRCB010−) and those induced by *Pseudomonas stutzeri* NRCB010 (NRCB010+).

No.	Retention Time	Compounds	Relative Area (%)	
	(min)		NRCB010−	NRCB010+
1	7.973	12,15-Octadecadiynoic acid, methyl ester	0.35 **	0.16
2	9.599	Nonanal dimethyl acetal	0.33	0.44 *
3	10.425	Octaethylene glycol monododecyl ether	0.22	0.30 *
4	10.544	3,4-Dimethylbenzamide	1.96	3.00 *
5	11.275	Octadecane, 6-methyl-	0.37 *	0.28
6	12.071	Heptadecane, 2,6,10,14-tetramethyl-	0.75 **	0.46
7	12.738	2,4-Di-tert-butylphenol	6.23	7.60 *
8	17.248	Methyl hexadecanoate	8.50	13.59 **
9	17.503	n-Hexadecanoic acid	0.51	0.88 **
10	18.928	Ethyl iso-allocholate	0.24	0.31 **
11	19.152	Methyl stearate	5.24	11.45 *

Note: The asterisks after the data of the same component mean significant differences between treatments by *t* test. * *p* < 0.05, ** *p* < 0.01.

**Table 4:** Relationship between tomato root exudate metabolite relative content and biomass, diameter of swarming area, and biofilm formation of *Pseudomonas stutzeri* NRCB010.

No.	Metabolite	Biomass (OD600)	Diameter of Swarming Area (cm)	Biofilm Formation (OD560)
1	12,15-Octadecadiynoic acid, methyl ester	−0.803	−0.929 **	−0.898 *
2	Nonanal dimethyl acetal	0.648	0.931 **	0.857 *
3	Octaethylene glycol monododecyl ether	0.525	0.643	0.457
4	3,4-Dimethylbenzamide	0.720	0.934 **	0.963 **
5	Octadecane, 6-methyl-	−0.660	−0.572	−0.492
6	Heptadecane, 2,6,10,14-tetramethyl-	−0.741	−0.894 *	−0.870 *
7	2,4-Di-tert-butylphenol	0.548	0.969 **	0.901 *
8	Methyl hexadecanoate	0.816 *	0.841	0.784
9	n-Hexadecanoic acid	0.678	0.973 **	0.891 *
10	Ethyl iso-allocholate	0.812 *	0.841 *	0.788
11	Methyl stearate	0.673	0.969 **	0.965 *

Note: Pearson correlations between tomato root exudate metabolite relative content and biomass, diameter of swarming area, and biofilm formation were performed using SPSS version 26.0. * *p* < 0.05, ** *p* < 0.01.

## Data Availability

The data presented in this study are contained within the article.

## Supplementary Materials

The following supporting information can be downloaded at: https://www.mdpi.com/article/10.3390/metabo13050664/s1, Figure S1: Effects of n-hexadecanoic acid (Hac) on *Pseudomonas stutzeri* NRCB010 responding to (A) NaCl, (B) pH, and (C) Cu^2+^ stresses; Figure S2: Ion chromatogram of root exudates of tomato (A), and tomato inoculated with NRCB010 (B); Table S1: Compositions of tomato root exudates (RE/ NRCB010−) and induced by *Pseudomonas stutzeri* NRCB010 (RE/ NRCB010+).
