# Supplementary material for: Certain Tomato Root Exudates Induced by Pseudomonas stutzeri NRCB010 Enhance Its Rhizosphere Colonization Capability
Source: Metabolites. 2023 May 16;13(5):664. doi: 10.3390/metabo13050664 (PMC10220591; doi:10.3390/metabo13050664)
Supplement: Supplementary file 1 [file metabolites-13-00664-s001.zip › metabolites-2342180-supplementary.pdf]

Supplementary materials

Supplementary methods

Fifty mg L^{-1} n-hexadecanoic acid on *Pseudomonas stutzeri* NRCB010 responding to NaCl, pH, and Cu^{2+} stresses were evaluated with a 96-well microplate. 188 μL one-fifth NBNS medium with different pH, NaCl, and Cu^{2+}, 10 μL NRCB010 suspension, and 2 μL 5 g L^{-1} n-hexadecanoic acid were added into a well, mixed thoroughly, and then incubation at 30℃, 100 rpm shaker for 48 h. OD600 was measured by a microplate analyzer to indicating NRCB010 biomass.

Supplementary figures

Figure S1.

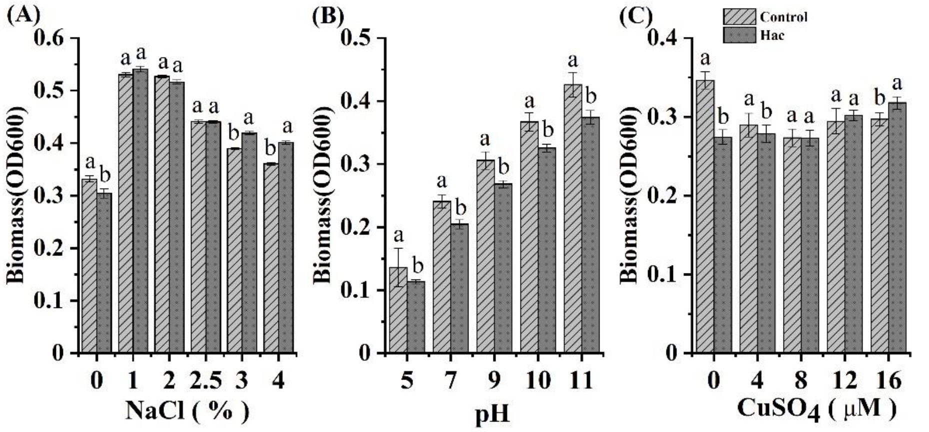

Figure S1. Effects of n-hexadecanoic acid (Hac) on *Pseudomonas stutzeri* NRCB010 responding to (A) NaCl, (B) pH, and (C) Cu^{2+} stresses. Bars are means ± standard error (n = 16). Different letter above the bars at the same stress's condition denote significant differences between treatments by Duncan's post-hoc test ($p < 0.05$)

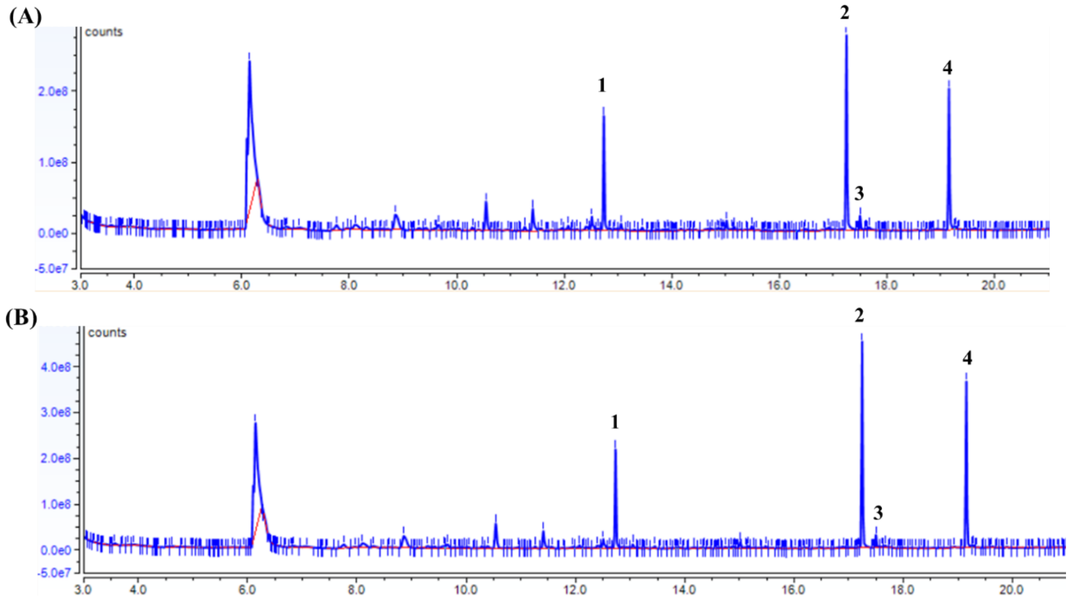

Figure S2. Ion chromatogram of root exudates of tomato (A), and tomato inoculated with NRCB010 (B). 1. 2,4-Di-tert-butylphenol (12.738 min), 2. Methyl hexadecanoate (17.248 min), 3. n-Hexadecanoic acid (17.503 min), 4. Methyl stearate (19.152 min).

Supplementary table

Table S1 Compositions of tomato root exudates (RE/ NRCB010−) and induced by *Pseudomonas stutzeri* NRCB010 (RE/ NRCB010+)

No.	Retention Time (min)	Compounds	Relative area (%)	
			RE/ NRCB010−	RE/ NRCB010+
1	3.572	t-Butyl-(2-[3-(2,2-dimethyl-6-methylene-cyclohexyl)-propyl]- [1,3] dithian-2-yl)-dimethyl-silane	0.24	0.29
2	4.599	Cystine	0.16	0.13
3	6.150	2-Pyrrolidinone, 1-methyl-	29.11	23.27
4	6.850	Mannosamine	0.21	0.29
5	7.776	Aziridine, 2-methyl-2-phenyl-	0.76	0.75
6	7.973	12,15-Octadecadiynoic acid, methyl ester	0.35 **	0.16
7	8.160	2,6-Xylidine	0.62	1.14
8	8.323	6,9,12-Octadecatrienoic acid, phenylmethyl ester, (Z, Z, Z)-	0.37	0.38
9	8.605	Dodecanoic acid, 3-hydroxy-	0.16	0.17
10	8.864	Benzaldehyde, 3,5-dimethyl-	2.88	2.75
11	9.303	Pterin-6-carboxylic acid	0.47	0.38
12	9.432	Octadecane, 6-methyl-	0.50	0.26
13	9.599	Nonanal dimethyl acetal	0.33	0.44 *
14	9.663	2,3,5,8-tetramethyl-Decane	0.89	0.56
15	9.980	l-Gala-l-ido-octose	0.24	0.30
16	10.299	Octadecane, 6-methyl-	0.48	0.29
17	10.425	Octaethylene glycol monododecyl ether	0.22	0.30 *
18	10.544	3,4-Dimethylbenzamide	1.96	3.00 *
19	10.918	5-Dimethyl (dichloromethyl) silyloxypentadecane	0.20	0.22
20	11.275	Octadecane, 6-methyl-	0.37 *	0.28
21	11.418	Dodecanal	1.70	1.86
22	11.578	Octadecane, 6-methyl-	0.40	0.28
23	11.952	Octadecane, 6-methyl-	0.41	0.26
24	12.071	Heptadecane, 2,6,10,14-tetramethyl-	0.75 **	0.46
25	12.401	Tetradecane, 2,6,10-trimethyl-	0.80	0.50
26	12.517	2,6-dimethyl-Heptadecane	1.47	0.96
27	12.738	2,4-Di-tert-butylphenol	6.23	7.60 *
28	12.857	Cyclopropanedodecanoic acid, 2-octyl-, methyl ester	0.20	0.16
29	13.058	Eicosane, 2-methyl-	0.61	0.41
30	13.445	Dodecane, 1,1-dimethoxy-	0.29	0.32
31	14.741	Octadecane, 6-methyl-	0.33	0.22
32	15.020	Decane, 2,3,5,8-tetramethyl-	0.79	0.50
33	15.163	Tetradecanoic acid, 12-methyl-, methyl ester	0.27	0.24
34	15.489	Heptadecane, 2,6,10,15-tetramethyl-	0.44	0.28
35	17.248	Methyl hexadecanoate	8.50	13.59 **
36	17.503	n-Hexadecanoic acid	0.51	0.88 **
37	17.670	Heptadecane, 2,6,10,15-tetramethyl-	0.35	0.32

38	18.928	Ethyl iso-allocholate	0.24	0.31 **
39	19.152	Methyl stearate	5.24	11.45 *
40	19.282	Tetradecane, 2,6,10-trimethyl-	0.29	0.20
41	19.646	2-Myristynoyl pantetheine	0.25	0.20

Note: the more asterisks after the data of the same component means more significant differences between RE/ NRCB010− and RE/ NRCB010+ treatments by *t* test. *$p < 0.05$, ** $p < 0.01$.
